# Insomnia Promotes Hepatic Steatosis in Rats Possibly by Mediating Sympathetic Overactivation

**DOI:** 10.3389/fphys.2021.734009

**Published:** 2021-09-24

**Authors:** Zongding Wang, Xiaoyan Liang, Yanmei Lu, Tiemin Jiang, Tuerganaili Aji, Kalibixiati Aimulajiang, Huaxin Sun, Ling Zhang, Xianhui Zhou, Baopeng Tang, Hao Wen

**Affiliations:** ^1^ State Key Laboratory of Pathogenesis, Prevention, and Treatment of High Incidence Diseases in Central Asia, The First Affiliated Hospital of Xinjiang Medical University, Urumqi, China; ^2^ Hepatobiliary and Hydatid Disease Department, First Affiliated Hospital of Xinjiang Medical University, Urumqi, China; ^3^ Department of Pacing and Electrophysiology, The First Affiliated Hospital of Xinjiang Medical University, Xinjiang, China; ^4^ Xinjiang Key Laboratory of Cardiac Electrophysiology and Cardiac Remodeling, The First Affiliated Hospital of Xinjiang Medical University, Xinjiang, China

**Keywords:** insomnia, sleep deprivation, sympathetic nerve, hepatic steatosis, lipid droplets

## Abstract

**Background:** Insomnia is a widespread problem that can lead to the occurrence of other diseases and correlates closely with sympathetic nerve hyperactivation. Obesity-induced hepatic steatosis is mediated by sympathetic overactivation. However, it remains unclear whether insomnia may cause hepatic steatosis. The goal of this study was to preliminarily investigate whether insomnia caused hepatic steatosis in rats *via* sympathetic hyperactivation.

**Methods:** A total of 32 Sprague-Dawley male rats were divided randomly into four groups: model, sympathetic denervation (Sd), estazolam, and control (eight rats/group). Model group received sustained sleep deprivation using the modified multiple platform method. In the Sd group, rats underwent sleep deprivation after receiving Sd by 6-hydroxydopamine (6-OHDA). Estazolam group: the rats concurrently received sleep deprivation and treatment with estazolam. The other eight rats housed in cages and kept in a comfortable environment were used as control. Blood samples were obtained for analysis of plasma lipids and hepatic function. Sympathetic hyperactivation-related indexes and hepatic steatosis in liver tissues were tested.

**Results:** Liver enzymes, plasma lipid levels, and hepatic steatosis were elevated in insomnia rats, and sympathetic hyperactivation was found. Insomnia-induced hepatic steatosis was effectively lowered with pharmacological ablation of the hepatic sympathetic nerves. Furthermore, the treatment of insomnia with estazolam inhibited sympathetic activation and reduced hepatic steatosis.

**Conclusion:** Sustained sleep deprivation-induced insomnia promotes hepatic steatosis in rats possibly by mediating sympathetic overactivation.

## Introduction

Insomnia is a common problem that warrants increased attention from clinicians (Buysse, 2013). Previous studies indicated a high incidence of sleep disturbance in the general population, including insomnia (Kalmbach et al., 2019), hypersomnia (Belenky et al., 2003), circadian rhythm sleep disorders, and other sleep dysfunction (Kammerer et al., 2021), especially chronic insomnia, which is highly correlated with increased morbidity and mortality (Lu et al., 2018). People with long-term insomnia are prone to nonrestorative or poor-quality sleep and even suffering from depression, anxiety (Okun et al., 2018), cognitive impairment (Fortier-Brochu and Morin, 2014), mental disorder (Medrano-Martinez and Ramos-Platon, 2016), concomitant cardiovascular, and other diseases if left untreated (Jarrin et al., 2018). Some studies have suggested an association of insomnia with increased sympathetic activity (Carter et al., 2018). The possible mechanism might be correlated with dysregulation of the hypothalamic-pituitary-adrenal axis (van Liempt et al., 2013). In addition, chronic insomnia is often accompanied by impairment of the sympathetic baroreflex (Carter et al., 2018). Therefore, sympathetic hyperactivation may be closely related to the development of insomnia.

The liver is the major organ responsible for lipid metabolism, modulating hepatic lipid metabolism and homeostasis (Jensen-Cody and Potthoff, 2021). Its main functions include lipid synthesis, metabolism, and transportation. Lipids mainly include triglycerides (TGs), phospholipids, and cholesterol (Schulze and McNiven, 2019). Dysregulation of hepatic lipid metabolism leads to abnormal accumulation of lipids and the formation of lipid droplets in liver tissues, ultimately predisposing patients to fatty liver. Furthermore, patients with fatty liver are increasingly susceptible to steatohepatitis and cirrhosis compared with those nonfatty liver (Gluchowski et al., 2017). Thus, fatty liver is gaining increasing attention.

The liver receives innervation from the autonomic nervous system, including the sympathetic and parasympathetic nervous systems (Furness et al., 2014). Sympathetic nerves, as regulators, are involved in hepatic lipid metabolism (Amir et al., 2020). Sympathetic hyperactivation promoted hepatic steatosis. In contrast, sympathetic denervation could ameliorate high-fat diet-induced fatty liver in obese mice (Hurr et al., 2019). These results provide clues for sympathetic nerves to regulate hepatic lipid metabolism.

However, the hepatic sympathetic nerve activity of chronic insomnia is involved in lipid metabolism is unknown. Here, a chronic insomnia model was established. The present study aimed to validate the hypothesis that hepatic sympathetic nerve activity mediates hepatic steatosis in rats with chronic insomnia.

## Materials and Methods

### Animals and Welfare

Sprague-Dawley (SD) male rats (180–220g; 6–8weeks) were purchased from our institution and randomly divided into four groups (eight rats per group). The four groups were the control, model group, sympathetic denervation group (Sd), and treatment group (estazolam). The present study protocol was reviewed and approved by the Institutional Animal Ethics Committee of Xinjiang Medical University (IACUC-20210301-01), in accordance with the 588th regulation of animal experiments issued by the Chinese Government in 2011. Rats were euthanized by anesthetic overdose. Blood samples and liver specimens were obtained for analysis.

### Intraperitoneal Administration of 6-Hydroxydopamine

The sympathetic denervation group received 6-hydroxydopamine (6-HODA; #28094-15-7, Nanjing Chemical Co., Ltd.). Rats were injected intraperitoneally once daily for 3 consecutive days (50mg/kg/day; Chen et al., 2016; Hurr et al., 2019). Subsequently, these rats received sleep deprivation.

### Animal Model Establishment

Multiple modified platforms were used to establish the sleep deprivation model (Alkadhi and Alhaider, 2016). Except for eight rats in the normal control group, the remaining rats were prepared to establish the chronic insomnia model. In brief, rats were placed on a small fixed platform surrounded by water for 8weeks to sustainably maintain sleep deprivation and had access to water and food only. The normal control rats were housed in cages and kept in a comfortable environment under a 12-h light/dark cycle with water and food available *ad libitum*. All rats were fed with common pellet diets, and each group were given the same total amount of food every day. The treated rats received combined sleep deprivation and 5mg/kg/day disulfiram by estazolam (Hirase et al., 2008). The schematic of the experimental timeline was delineated in the Supplementary Figure 1.

### Hepatic Function and Lipid Level Assay

Blood samples were centrifuged, and the supernatant was collected for hepatic function tests. The levels of aspartate transaminase (ALT) and alanine aminotransferase (AST) were determined by an automatic biochemical analyzer (Mindray-120, Guangzhou, China) in accordance with the instructions. Free fatty acids (FFAs) and TGs were measured by kits with a colorimetric method (Nanjing Jianchen Biotech Inc., Nanjing, China).

### Histological Analysis and Immunohistochemistry

Paraffin-embedded liver sections were deparaffinized, baked, dewaxed and hydrated. Hematoxylin-eosin (HE) staining was performed according to routine protocols. For oil red O staining, the cardiac tissue was sliced using a freezing microtome, rinsed, kept in 60% isopropanol, and finally stained with 0.5% oil red O solution (Sigma, St. Louis, United States) for 20min or maintained in 60% isopropanol for differentiation.

The process of immunohistochemical staining was performed following the manual instructions. In brief, endogenous peroxidase activity was quenched using 3% hydrogen peroxidase, samples were subjected to heat-mediated antigen retrieval (citrate buffer, pH6), and samples were then blocked with goat serum for 30min. Primary antibody incubation was performed overnight at 4°C using different dilutions, including Perilipin-2 (1:500, OriGene, Rockville, United States) and growth-associated protein-43 (GAP43; 1:1,000, Abcam, Cambridge, United States), and the secondary antibody was incubated for 1h at room temperature. The sections were washed three times (5min/wash) after each step. Substitution of PBS for the primary antibody served as the negative control. Diaminobenzidin (DAB; ZSGB-BIO, Beijing, China) was used to stain the sections. Then, sections were counterstained with hematoxylin. The stained sections were examined by a Leica DMI 3000R microscope (Leica Microsystems, Germany) and images were captured under an Olympus microscope (Olympus, DP26, Japan). Immunostaining was quantified using ImageJ software.

### Immunofluorescence Assay

Liver tissues were fixed in 4% formaldehyde over 24h, dehydrated, and embedded in paraffin. Sections (5μm) were deparaffinized and sequentially incubated overnight at 4°C with primary antibodies against GAP43 (1:100, Abcam, Cambridge, United States) and TH (1:50, Bioss, Beijing, China), washed and incubated for 1h with appropriate conjugated secondary antibodies (Alexa Fluor 488-conjugated donkey anti-mouse IgG and CoraLite 594-conjugated goat anti-rabbit IgG, Proteintech, United States). Nuclei were stained with 4', 6-diamidino-2-phenylindole (DAPI). Confocal microscopy imaging and colocalization analysis were carried out on a confocal laser scanning microscope (Leica, Germany).

### ELISA

After weighing and mashing, fresh liver tissues were lysed in PBS buffer supplemented with protease inhibitors (Mini EDTA-free Protease Inhibitor, Roche, Switzerland) and broken by sonication. After centrifugation (5,000×*g*, 10min), the supernatant was used for nerve growth factor (NGF) assays according to the manufacturer’s protocol (Jianglai Biotechnology Co., Shanghai, China). Absorbance at 450nm was measured using a microplate reader (Bio-Rad, Hercules, United States). Each measurement was performed in triplicate.

### Transmission Electron Microscopy

The subcellular organelles in liver tissues were observed by Transmission Electron Microscopy (TEM, JEM-1220, JEOL Ltd., Japan) and images were captured under an Olympus microscope (Olympus Soft Imaging Solutions, Morada G3, Japan). The method for liver preprocessing was in line with those from previous studies (Du et al., 2017). Briefly, the liver tissue was fixed with 4% glutaraldehyde for 24h, dehydrated by an ethanol gradient series, postfixed in 0.5% osmium tetroxide, 2% uranyl acetate, and 0.1% tannic acid, followed by epoxy resin fixation.

### High-Performance Liquid Chromatography-Tandem Mass Spectrometry

In this study, high-performance liquid chromatography-tandem mass spectrometry (HP-LC-MS/MS) was used to test the norepinephrine (NE) content in the liver. First, 10.0mg of NE (Zhongshan Golden Bridge, Beijing, China) was added to 0.1% ascorbic acid solution (1mg ascorbic acid dissolved in 1ml of methanol). The pretreatment of samples was conducted according to a previous study (Tsunoda et al., 2002). Then, the 250-μl sample was dried with a nitrogen gun and reconstituted with 50μl of acetonitrile: water (85:15) solution (containing 2% formic acid), and 20μl of sample and NE standard were added to the LC/MS vial. High-performance liquid chromatography (ACQUITY UPLC, Waters, United States) and mass spectrometry (ACQUITY TQD, Waters, United States) were used for analysis. The MS conditions were as follows: cone gas flow rate of 150L/h, desolvation gas flow rate of 900L/h, source temperature of 150°C, desolvation temperature of 550°C, and ionization mode ESI+. Finally, the samples were subjected to gradient elution as follows: mobile phase A (5:95 acetonitrile containing 30mM formic acid: Milli-Q water) and mobile phase B (85:15 acetonitrile containing 30mM formic acid: Milli-Q water).

### Statistical Analysis

The data are presented as the mean±SD, comparisons between two groups were carried out using the independent T test and among multiple groups using single factor ANOVA. About *p*<0.05 was considered statistically significant. All statistical calculations were performed using the statistical software program (GraphPad Prism 8.0 software, United States).

## Results

### Sustained Sleep-Deprivation Occurred in the Model Group

After receiving 8weeks of sleep deprivation, the mental state, activity, and hair luster of the rats were selected as the main observation indexes in the present experiment. The rats in the model group showed obvious retarded reactions, reduced activity, and dull hair. The rats in the treatment group and the sympathetic denervation group exhibited shiny fur and improvements in the previously mentioned symptoms. However, no abnormal findings were observed in the control group.

### Liver Enzymes and Lipid Metabolism Tests Were Abnormal in Insomnia Rats

**Figure 1:**
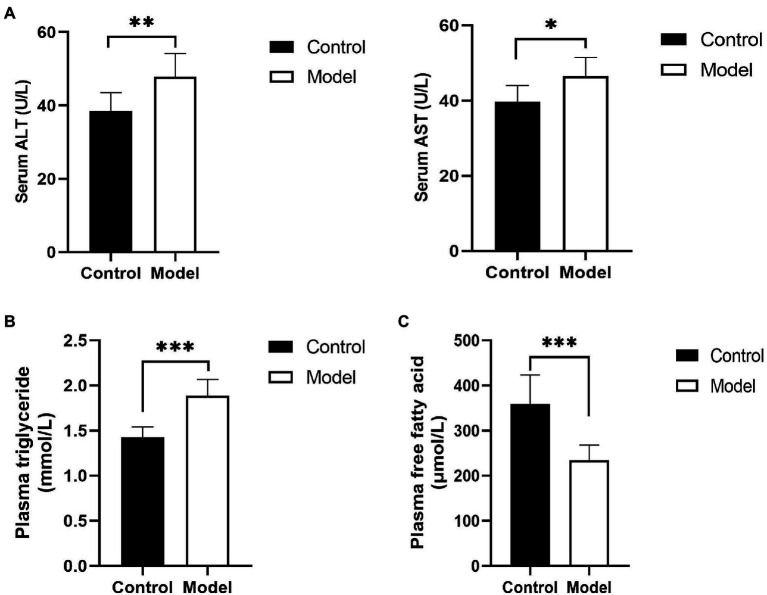
Changes in hepatic function and blood lipids. **(A)** Serum was analyzed for liver enzyme [aspartate transaminase (ALT) and alanine aminotransferase (AST)] levels; **(B)** plasma triglyceride levels in insomnia rats were clearly higher than those in controls; and **(C)** Plasma free fatty acid (FFA) levels in insomnia rats were significantly lower than those in controls. The results were obtained from two independent experiments and are expressed as the mean±SD, *n*=8 per group, ^*^ *p*<0.05; ^**^ *p*<0.01; and ^***^ *p*<0.001.

To determine the change in hepatic function of the insomnia rats, serum ALT, and AST were detected. Compared with the control group, the rats in the model group displayed a higher level of liver enzymes (ALT, 47.8±6.3 vs. 38.5±4.9U/L, *p*=0.0058; AST, 46.4±5.0 vs. 39.7±4.3U/L, *p*=0.0124U/L; Figure 1A). Moreover, to evaluate insomnia-induced plasma lipid changes, we analyzed the plasma lipids of the control and model groups. The level of triglycerides in the model group was significantly higher than that in the control group (1.88±0.18 vs. 1.42±0.11mmol/L, *p*<0.0001; Figure 1B). Conversely, a greater reduction in the free fatty acid content was observed in the model group (233.95±33.92 vs. 359.51±63.90μmol/L, *p*=0.0002; Figure 1C).

**Figure 2:**
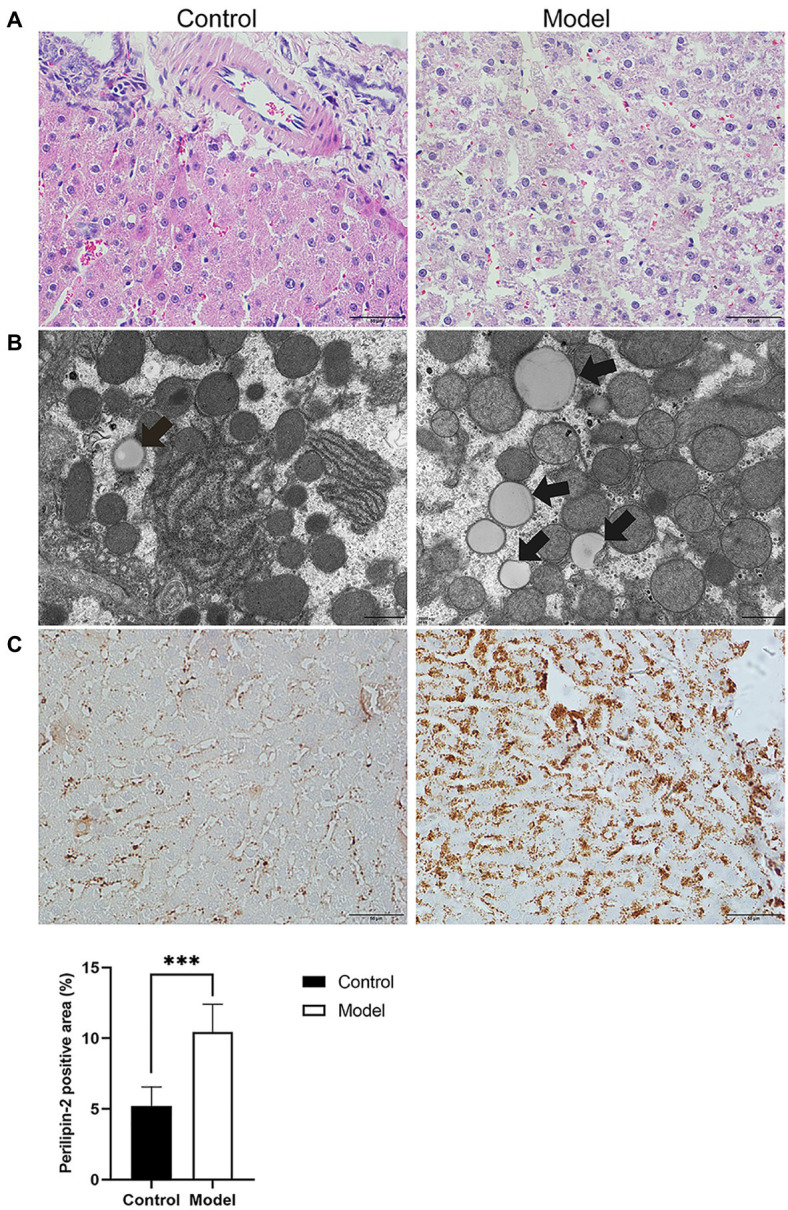
Histopathology, immunohistochemistry, and transmission electron microscopy (TEM) were performed to determine the degree of hepatic steatosis. **(A)** Hematoxylin-eosin (HE) staining of the liver showed steatosis of most hepatocytes in the model group (scale bar: 50μm). **(B)** Lipid droplets in hepatocytes were examined by TEM (scale bar: 1μm). **(C)** Immunohistochemical staining for Perilipin-2 was observed predominantly in hepatocytes, where positive staining was brown-yellow (scale bar: 50μm). The results were obtained from two independent experiments and are expressed as the mean±SD, *n*=8 per group, ^***^ *p*<0.001.

To further investigate the effects of insomnia on fatty liver, we inspected the pathology and microstructure of the liver. HE staining of the liver in the model group indicated that more lipid droplets accumulated in the hepatocytes of the model group than in the hepatocytes of the control rats (Figure 2A). Additionally, the subsequent results from electron microscopy also supported this conclusion (Figure 2B). Perilipin-2, which is expressed around lipid droplets, was assessed to determine the extent of fat accumulation in the liver. The immunohistochemistry results revealed that compared to the control group, the expression of Perilipin-2 was significantly increased in the model group (10.45±1.95 vs. 5.21±1.34%, *p*<0.0001; Figure 2C).

### Sympathetic Activation and Hyperplasia in the Livers of the Model Group

**Figure 3:**
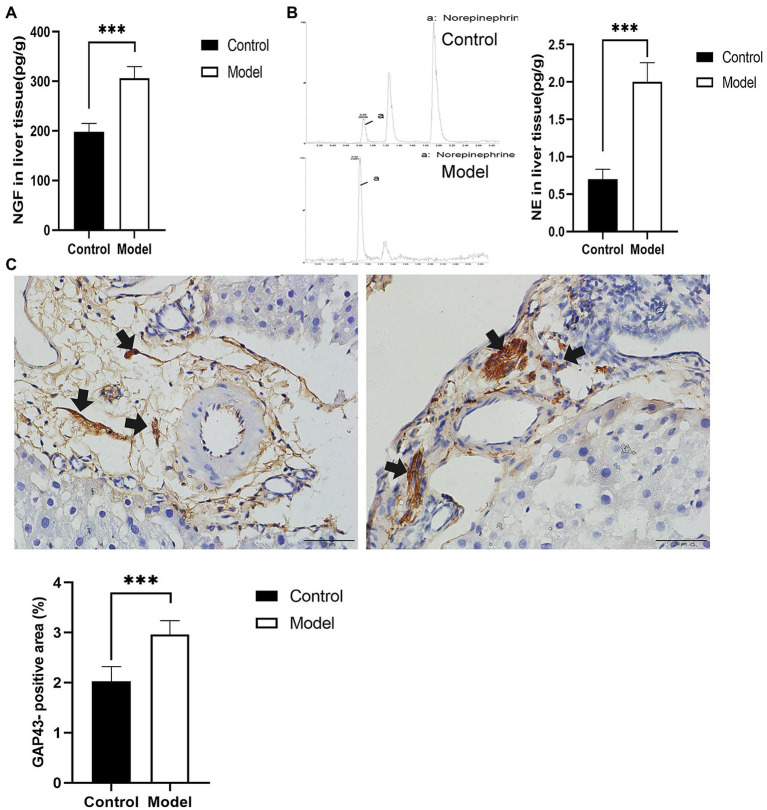
Determination of nerve growth-related indexes in liver tissue. **(A)** Nerve growth factor (NGF) was determined by ELISA in liver tissues. **(B)** Norepinephrine (NE) in liver tissues was quantified by high-performance liquid chromatography-tandem mass spectrometry (HP-LC-MS/MS), presenting an increase in the model group. **(C)** Immunohistochemical staining of growth-associated protein-43 (GAP43) was used to assess nerve growth (scale bar: 50μm). The results were obtained from two independent experiments and are expressed as the mean±SD, *n*=8 per group, ^***^ *p*<0.001. NE, norepinephrine.

**Figure 4:**
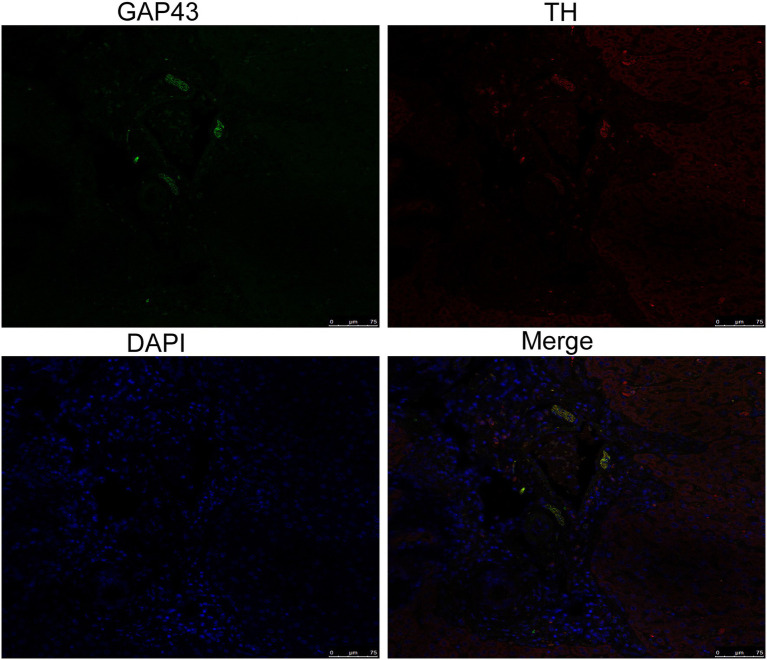
Identification of sympathetic nerves in the liver by confocal laser scanning microscopy. GAP43/TH colocalization indicated hyperplastic sympathetic nerves in the livers of insomnia rats (scale bar: 75μm).

To explore the effect of insomnia on the growth of the sympathetic innervation status in the liver. We first examined the levels of NGF and NE in hepatic tissue. Our results showed that the levels were significantly elevated in the model group compared with the control group (NGF, 306.24±23.64 vs. 197.98±17.13pg/g, *p*<0.0001; NE, 2.00±0.29 vs. 0.70±0.12pg/g, *p*<0.0001; Figures 3A,B). Immunohistochemistry for GAP43, an indicator of neuronal growth, was performed to investigate axonal regeneration in liver tissue, which suggested more active nerve sprouting in the model group (0.54±0.08 vs. 2.02±0.25%, *p*<0.0001; Figure 3C). To identify these nerves, immunofluorescence was used to colocalize GAP43 with TH in the liver tissue. These observations suggested active and hyperplastic sympathetic nerves in the livers of the model group (Figure 4).

### Sympathetic Denervation Improved Hepatic Steatosis

**Figure 5:**
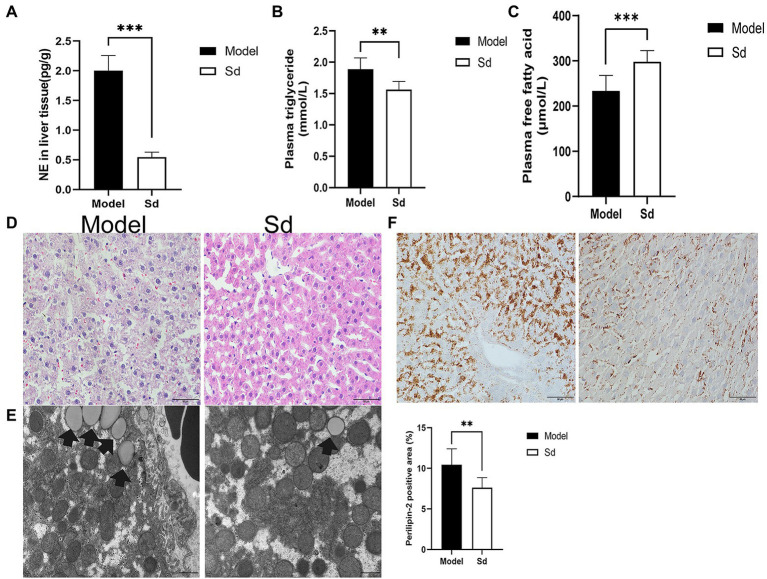
The effect of sympathetic denervation on hepatic steatosis. **(A)** NE of sympathetic neuromediators in liver was detected. **(B)** The plasma triglyceride levels in the liver tissues were analyzed. **(C)** The plasma free fatty acid levels were detected. **(D)** HE staining (scale bar: 50μm). **(E)** TEM was performed to observe lipid droplets (scale bar: 1μm). **(F)** Quantitative analysis of lipid droplets was measured by Perilipin-2 immunohistochemistry (scale bar: 50μm). The results were obtained from two independent experiments and are expressed as the mean±SD, *n*=8 per group, ^**^ *p*<0.01; and ^***^ *p*<0.001. Sd, sympathetic denervation group; NE, norepinephrine.

Injection of 6-OHDA is commonly used as a model of sympathetic denervation. To determine the effects of denervation, NE was measured in hepatic tissue after administration. In comparison with the model group, the NE content in the sympathetic denervation group evidently decreased (2.00±0.25 vs. 0.70±0.12pg/g, *p*<0.0001; Figure 5A).

In addition, the plasma levels of FFAs and TGs were analyzed, as well as the protein levels of Perilipin-2 in the liver tissues. Compared with the model group, TGs and Perilipin-2 levels decreased significantly in the 6-OHDA administration group, while FFAs showed the opposite trend (FFAs, 298.01±24.44 vs. 233.95±33.92μmol/L, *p*=0.0007; TGs, 1.56±0.13 vs. 1.88±0.18mmol/L, *p*=0.001; Perilipin-2: 7.61±1.23 vs. 10.45±1.95%, *p*=0.0038; Figures 5B,C,F). Similar results were obtained by HE staining and TEM images (Figures 5D,E). These results suggested that the sympathetic nerve was involved in insomnia-induced liver steatosis, exerting a promotion function.

### Estazolam Alleviated Fatty Liver in Insomnia Rats

**Figure 6:**
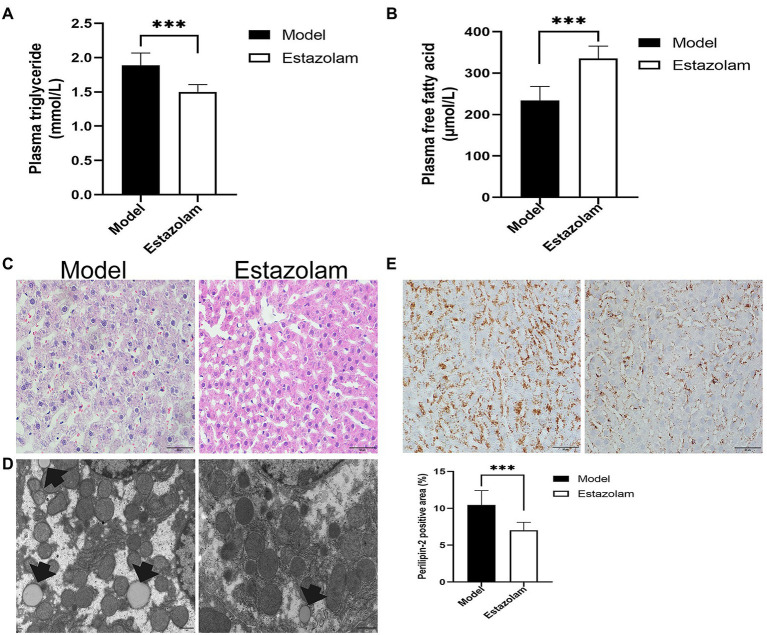
Blood lipids and hepatic steatosis were detected to evaluate the effect of estazolam on fatty liver in insomnia rats. **(A)** The plasma triglyceride levels. **(B)** The plasma free fatty acid levels. **(C)** HE staining (scale bar: 50μm). **(D)** Detection of lipid droplets by TEM (scale bar: 1μm). **(E)** Expression of Perilipin-2 was determined by immunohistochemistry (scale bar: 50μm). The results were obtained from two independent experiments and are expressed as the mean±SD, *n*=8 per group, ^***^ *p*<0.001.

Estazolam is the first-line therapy for insomnia. To evaluate the effect of estazolam on fatty liver in insomnia rats, the plasma FFAs and TGs levels were determined, as well as histopathological examinations. Compared with the model group, the plasma FFAs level was markedly elevated (335.65±29.41 vs. 233.95±33.92μmol/L, *p*<0.0001), while the TGs level was decreased in the treatment group (1.50±0.10 vs. 1.88±0.18mmol/L, *p*=0.0038; Figures 6A,B).

Subsequently, we investigated the change in Perilipin-2 in the liver after treatment. Immunohistochemical staining showed that hepatic steatosis was significantly decreased in the treatment group (7.03±1.06 vs. 10.45±1.95%, *p*=0.0007; Figure 6E). Moreover, the HE and microscopic tests were similar to the above immunohistochemical results (Figures 6C,D). It appears that supplementation with estazolam ameliorates fatty liver in insomnia rats.

### Estazolam Administration Inhibited Sympathetic Activation

**Figure 7:**
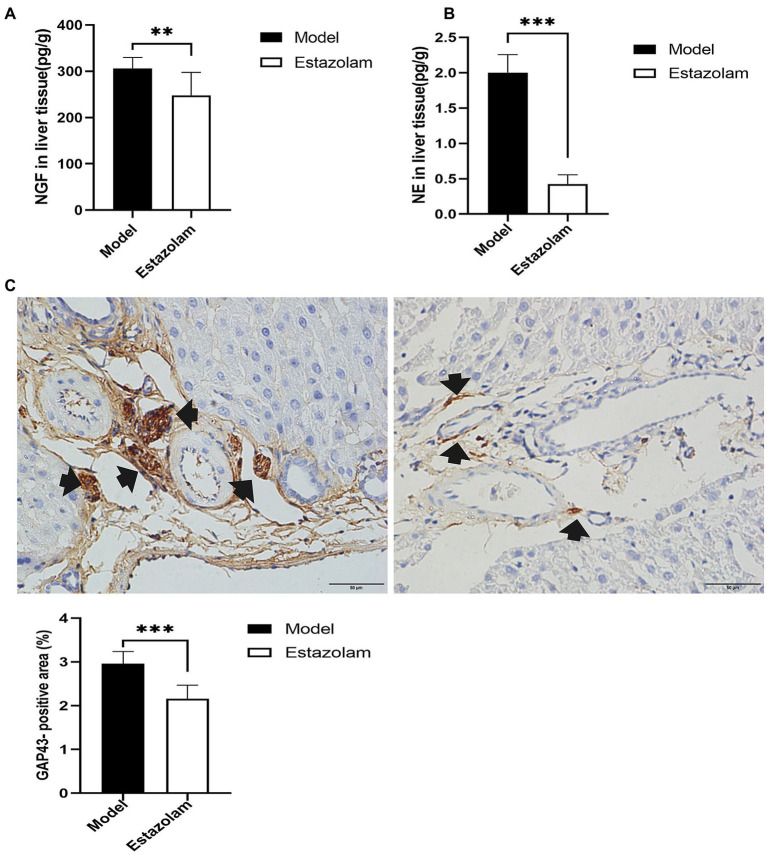
Sympathetic innervation in liver was evaluated. **(A)** The NGF level was tested in liver tissues. **(B)** NE in liver tissues was detected by HP-LC-MS/MS. **(C)** GAP43 staining was observed in the liver as a qualitative metric of neural growth by immunohistochemistry (scale bar: 50μm). The results were obtained from two independent experiments and are expressed as the mean±SD, *n*=8 per group, ^**^ *p*<0.01 and ^***^ *p*<0.001. NE, norepinephrine.

To further explore whether estazolam prevents sympathetic activation in fatty liver in insomnia rats, the growth of sympathetic innervation in the liver was evaluated. NGF and NE in liver tissues showed that estazolam administration could suppress the growth of sympathetic nerves in the treatment group compared to the model group (NGF, 248.26±49.23 vs. 306.24±23.64pg/g, *p*=0.0095; NE, 0.42±0.13 vs. 2.00±0.29pg/g, *p*<0.0001; Figures 7A,B). The immunohistochemistry results also suggested that GAP43 expression in the treatment group was significantly attenuated (2.16±0.30 vs. 2.96±0.27%, *p*<0.0001; Figure 7C).

## Discussion

Persistent insomnia can lead to an impairment in the quality of life and mental health (Fadeuilhe et al., 2021). Recently, insomnia has been gaining attention. In the present study, we first observed that sustained sleep deprivation contributed to fatty liver. To determine whether the sympathetic nerve was involved in insomnia-mediated hepatic steatosis, we performed sympathetic denervation experiments with 6-OHDA. We found that fatty liver can be alleviated by the use of 6-OHDA prior to suffering from sustained sleep deprivation. These initial results suggested that the sympathetic nerve has a facilitatory role in fatty liver associated with insomnia. This result is consistent with the results of previous studies (Hurr et al., 2019). We then assessed whether this phenomenon would also be seen in the heart muscle. No myocardial steatosis in insomnia rats was found in the results of Oil Red O staining. This result might correlate with the fact that the liver was the major tissue site for fat deposition and metabolism (Supplementary Figure 2).

Estazolam is an effective agent for insomnia control (Cohn et al., 1991). Based on the experimental data, there was an alleviation in the severity of fatty liver in the rats that received estazolam treatment. Furthermore, to understand the mechanisms underlying these experimental phenomena, we examined sympathetic activation, NGF and its neurotransmitter. Thus, these results offer preliminary evidence that estazolam inhibits sympathetic activation and relieves hepatic steatosis in insomnia rats. Besides, serum aminotransferase activity was measured in insomnia group and control group. The main purpose of this data was to evaluate the effects of sustained sleep deprivation on hepatic function only. Since both Sd group and estazolam group underwent pharmacological intervention, these drugs might produce liver enzyme abnormalities that would likely complicate the results shown in our study.

The modified multiple platform method was used to establish the sleep deprivation model, which is currently a widely used sleep deprivation rat model. The primary advantage of this method is its simplicity. When rats reached the sleep phase, muscle atonia caused them to lose balance on a platform and fall into the water and awaken (Machado et al., 2004). In our experiments, rats underwent sustained sleep deprivation for 8weeks, with the main aim of achieving complete insomnia. These rats subsequently developed unusual presentations, including retarded reactions, reduced activity, and dull hair. However, these results are not completely consistent with previous studies showing that rats increased their activity after suffering from short-term sleep deprivation.

In addition, the relationship between the sympathetic nerve and insomnia is complex and tightly linked. Sympathetic overactivation would cause insomnia, and insomnia in turn would exacerbate a range of physical and mental health problems (Taylor et al., 2016). The insomnia rat model was established by long-term sleep deprivation in our study, which was associated with sympathetic hyperactivity. Estazolam for insomnia has been widely employed. In one group of animals, eight rats received both estazolam therapy and sleep deprivation. The results indicated that estazolam was an efficacious pharmacological treatment for insomnia and suppressed sympathetic activation in the liver. These results also suggested that this agent is effective in reversing sympathetic hyperactivity in insomnia rats.

The liver plays a crucial regulatory role in fat metabolism (Alves-Bezerra and Cohen, 2017). Sympathetic overactivation in the insomnia model contributed to fatty liver. However, after treatment with 6-OHDA to destroy sympathetic nerves, hepatic steatosis significantly improved. These data suggested that sympathetic nerves are involved in the regulation of hepatic lipid metabolism. Furthermore, some studies have shown that obesity-induced hepatic steatosis is associated with hepatic sympathetic hyperactivity. Hepatic sympathetic denervation by 6-OHDA reduced obesity-induced hepatic steatosis (Hurr et al., 2019). This phenomenon coincided with our experimental conclusions.

From the clinical point of view, insomnia is a risk factor for nonalcoholic fatty liver disease (NAFLD; Kim et al., 2013). Recent studies have shown that insomnia is associated with metabolic disorders, including glucose metabolism and lipid metabolism (Mir et al., 2013; Seelig et al., 2013). Therefore, that is one reason short sleep duration is associated with diabetes, obesity, and fatty liver. Consistent results have also been found in a population-based observational study, showing that good sleep is associated with lower presence of NAFLD (Deng et al., 2021). However, the mechanisms for this result remain unclear. So, we conducted a preliminary exploration of this issue from the sympathetic nerve activity perspective.

In our research results, HE and microscopic tests were used to evaluate the degree of liver steatosis. In addition, Perilipin-2, which is expressed around lipid droplets, was used to directly assess lipid droplets by immunohistochemistry. These results could provide useful information for the evaluation of fatty liver. Hepatic steatosis is closely related to blood lipid levels. FFAs are a major secretory product of lipid droplets or adipocytes as a result of lipolysis of stored TGs. The major process of lipolysis involves the hydrolysis of TGs to FFAs and glycerol (Quiroga and Lehner, 2018). Therefore, TGs and FFAs levels were measured in the present study. These results indicated that TGs level were elevated as a result of insomnia and that FFAs levels were lower. In contrast, the opposite results were found in rats suffering insomnia treated with estazolam. These data implied that pharmacotherapy altered the metabolic states of synthesis or breakdown in insomnia rats. However, this requires further validation.

Regarding sympathetic nerve assessment, we first analyzed the expression of the nerve growth marker GAP43. The results suggested that hepatic autonomic nerves were in an active growth state in insomnia rats. Therefore, it was necessary to identify the sympathetic nerves using colocalization by fluorescence confocal microscopy. Further analysis was conducted on NGF and neurotransmitters in liver tissues. These results also provided preliminary evidence to support sympathetic overactivation.

However, our research still has some shortcomings and limitations. Firstly, pharmacological intervention for hepatic sympathetic denervation by 6-OHDA included intraperitoneal injection, which disrupted all sympathetic neurons in the abdominal cavity, thus lacking specificity. Secondly, the sample size in our initial exploratory study is small. In the future, we will expand the sample size to confirm these results. Thirdly, the relationship between insomnia, psychological stress, and sympathetic overactivation is intricate. To date, there have been few animal models of insomnia that can avoid the effects of psychological stress. Similarly, other mechanisms of benzodiazepines may also contribute to facilitation of falling asleep. Moreover, the detailed mechanisms that are involved in sympathetic nerve regulation of hepatic steatosis remain unclear, and further studies are warranted.

## Conclusion

In conclusion, sustained sleep deprivation-induced insomnia promotes hepatic steatosis in rats possibly by mediating sympathetic overactivation.

## Data Availability Statement

The original contributions presented in the study are included in the article/Supplementary Material, further inquiries can be directed to the corresponding authors.

## Ethics Statement

The animal study was reviewed and approved by Animal Ethics Committee of Xinjiang Medical University.

## Author Contributions

ZW and XL wrote the manuscript. All authors contributed to the article and approved the submitted version.

## Funding

This study was supported by the National Natural Science Foundation of China (project no.: 230805201133).

## Conflict of Interest

The authors declare that the research was conducted in the absence of any commercial or financial relationships that could be construed as a potential conflict of interest.

## Publisher’s Note

All claims expressed in this article are solely those of the authors and do not necessarily represent those of their affiliated organizations, or those of the publisher, the editors and the reviewers. Any product that may be evaluated in this article, or claim that may be made by its manufacturer, is not guaranteed or endorsed by the publisher.

## Supplementary Material

Supplementary Figure 1 Schematic presentation of experimental timeline and design. Experimental grouping rats were randomly divided into four groups, as indicated in the figure.

Supplementary Figure 2 Oil red O staining was performed to visualize myocardial steatosis in both control and insomnia rats (scale bar: 50μm). The results showed that the gross observations of myocardial steatosis from the two groups were no significant difference.
