# Supplementary figures and images for: Insomnia Promotes Hepatic Steatosis in Rats Possibly by Mediating Sympathetic Overactivation
Source: Front Physiol. 2021 Sep 24;12:734009. doi: 10.3389/fphys.2021.734009 (PMC8497715; doi:10.3389/fphys.2021.734009)

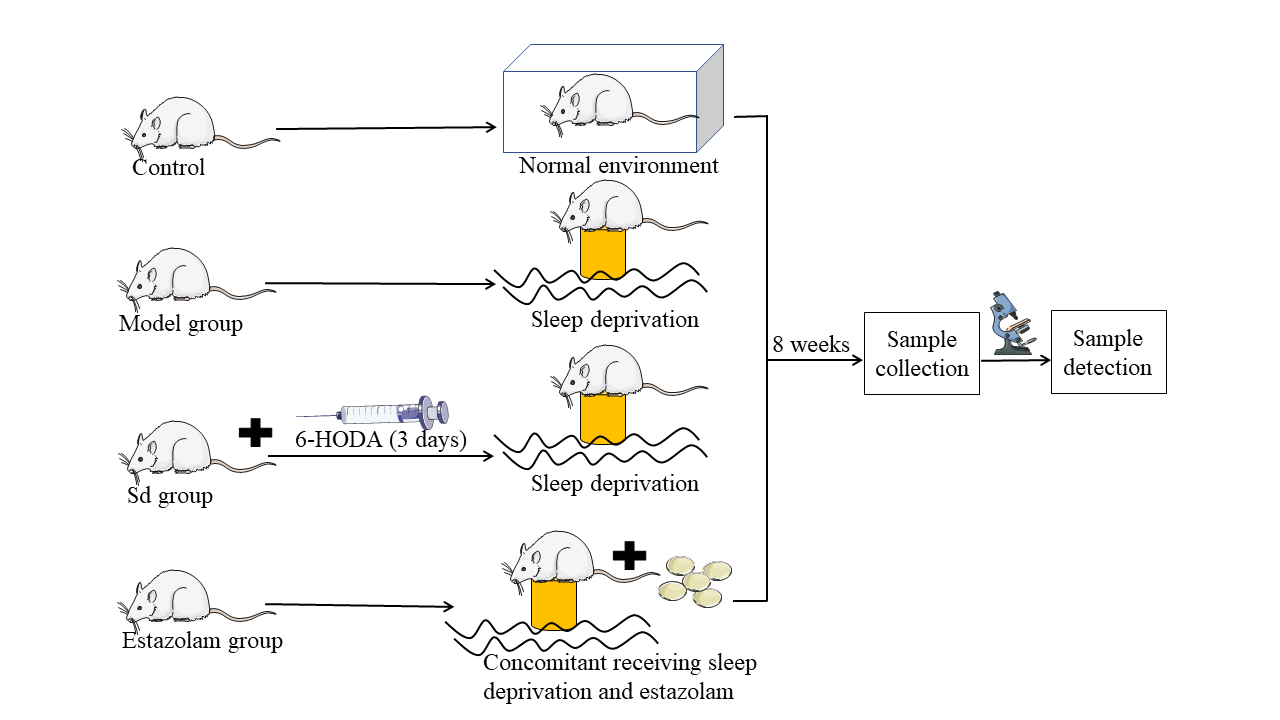

Supplement: Supplementary Figure 1 — Schematic presentation of experimental timeline and design. Experimental grouping rats were randomly divided into four groups, as indicated in the figure. [file Image_1.TIF]

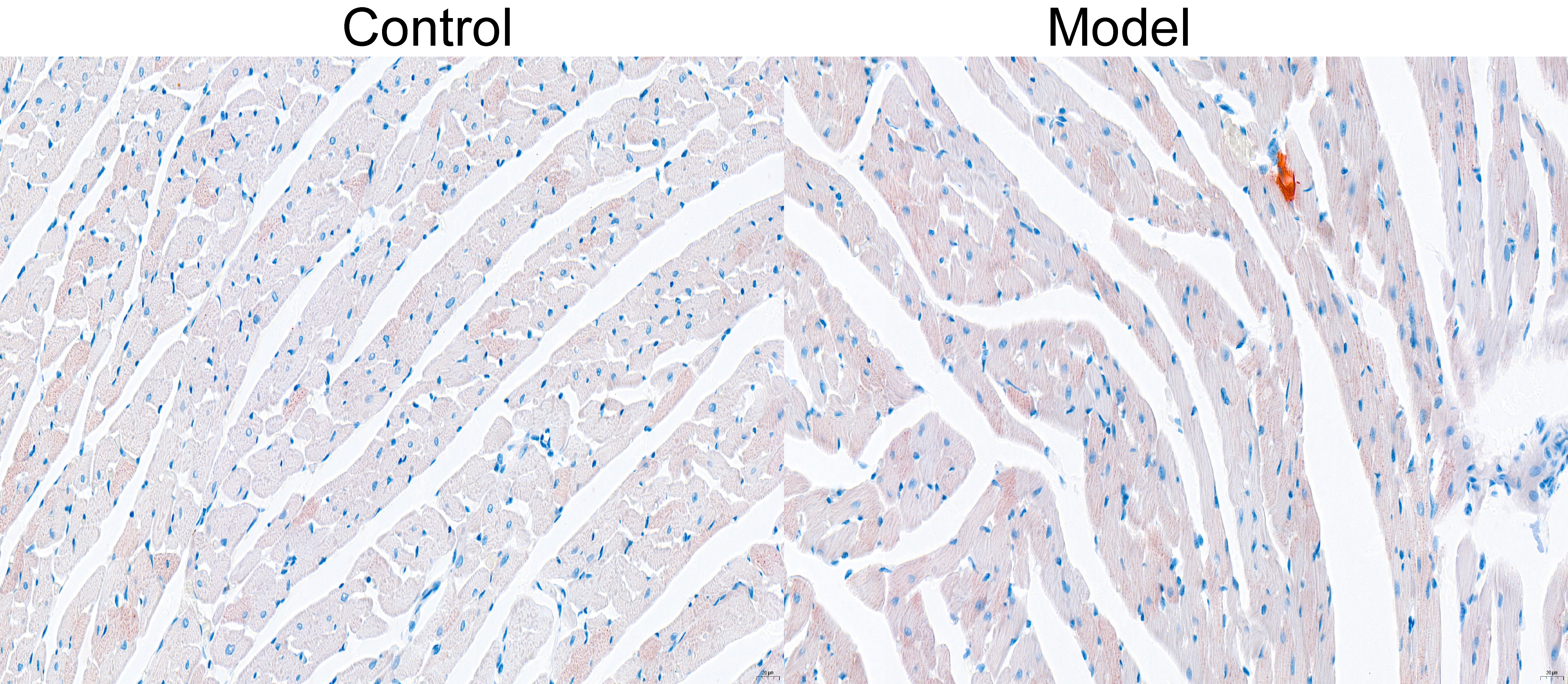

Supplement: Supplementary Figure 2 — Oil red O staining was performed to visualize myocardial steatosis in both control and insomnia rats (scale bar: 50μm). The results showed that the gross observations of myocardial steatosis from the two groups were no significant difference. [file Image_2.JPEG]
